# Differences in Response to Recombinant Growth Hormone Therapy on Height Gain in Patients with Idiopathic Short Stature Vs. Patients with Growth Hormone Deficiency

**DOI:** 10.7759/cureus.7319

**Published:** 2020-03-18

**Authors:** Ali K Alzahrani, Abdulmohsin K Algethami, Ghassan Barnawi, Ibraheem A Meftah, Ammar Alshanqiti, Hashim Al-Hashmi, Mohammed A Khan, Nailah Felimban

**Affiliations:** 1 Medicine, King Saud Bin Abdulaziz University for Health Sciences, Jeddah, SAU; 2 Brain Imaging, King Abdulaziz Medical City, Jeddah, SAU; 3 Medical Education, King Saud Bin Abdulaziz University for Health Sciences, Jeddah, SAU; 4 Endocrinology, King Abdulaziz Medical City, Jeddah, SAU

**Keywords:** growth hormone deficiency, idiopathic short stature, growth hormone therapy, recombinant human growth hormone

## Abstract

Objective

The use of recombinant human growth hormone (rhGH) in patients with idiopathic short stature (ISS) has been an area of concern since some studies reported less desired effects of the drug in this group of patients as compared to patients with growth hormone deficiency (GHD). In addition, there were no studies addressing the effects of rhGH in Saudi children. Therefore, we conducted a retrospective study to observe the effects one year of treatment with rhGH on the mean height gain in patients with ISS and GHD.

Methods

This retrospective study took place at King Abdulaziz Medical City in Jeddah. The study subjects included two groups of patients (GHD vs ISS). Patients' files were reviewed from January 2000 to January 2018 using the following parameters: chronological age, bone age, height, weight, body mass index (BMI), insulin-like growth factor (IGF-1), growth hormone stimulation test, and growth velocity (GV). After one year of treatment, the height, weight, and BMI of the study subjects were monitored and assessed.

Results

The total number of patients was 55, 36 of which were diagnosed with GHD while 19 were diagnosed with ISS. The mean age of patients with GHD and ISS were 10.7±2.38 and 10.91±2.74 years, respectively. Both groups showed a significant increase in height. The initial height for patients with GHD was 125.26±12.27 cm, and they achieved a mean height of 134.231±12.88 cm after one year of treatment. For the other group, the initial height for ISS patients was 125.51±10.94 cm, and they achieved a mean height of 134.04±10.90 cm after one-year therapy. However, after the treatment, there was no significant difference in the height gain between GHD and ISS patients (134.231±12.88, 134.04±10.90, respectively, P=0.437).

Conclusion

The short-term use of rhGH has a potent and similar effect on increasing the height of both patients diagnosed with ISS as well as GHD.

## Introduction

Growth hormone (GH) is a single-chain polypeptide protein synthesized, stored, and released from the anterior pituitary gland in response to a trigger hormone called GH-releasing hormone (GHRH) from the hypothalamus in the brain and moves within the bloodstream into the target cells. It is responsible for most of the metabolic and growth processes inside the body. The primary role of GH is mediated by another hormone called insulin-like growth factor1 (IGF-1), which is secreted from the liver. This hormone functions principally by stimulating cell division and growth. Therefore, any disturbance that affects GH secretion will lead to growth-related abnormalities. For example, excessive secretion of GH during childhood or adulthood can lead to gigantism or acromegaly, respectively. On the other hand, reduced production of this hormone can cause growth retardation and short stature [1-3]. Exogenous growth hormone treatment had been extracted from human pituitary glands and used to treat mainly those with severe GH deficiency. GH deficient patients are those with a peak growth hormone concentration of less than 10 ng/ml diagnosed by the growth hormone stimulation test. Since 1985, human-extracted GH was replaced by recombinant GH therapy (RGHT), which is used to treat a wider range of conditions. Recombinant GH is used to treat patients with GH deficiency and to improve growth and height gain in patients with idiopathic short stature (ISS). ISS is a condition in which the height is below two standard deviations (SD) of the mean for age in the absence of any endocrine, metabolic, or any other disease that explains the short stature [4-9].

Thus far, there are a few data and studies published on the long-term use of this medication in the treatment of patients with ISS and GHD. Only very few of them have compared the efficacy of RGHT between patients diagnosed with idiopathic short stature and patients with idiopathic GH deficiency. No studies have been conducted about the use of RGHT in Saudi Arabian children or compared the effect of RGHT between Saudi Arabian children with ISS versus. those with GHD. Therefore, we aim in this study to compare the effectiveness of RGHT in inducing height gain in Saudi patients with ISS versus GHD.

## Materials and methods

This retrospective study was performed by reviewing the medical records of patients with GHD and ISS at King Abdulaziz Medical City in Jeddah from January 2000 to January 2018. The diagnosis of GHD was through the growth hormone stimulation test. GH-deficient children were said to have a peak growth hormone concentration of less than 10 ng/ml on more than one occasion. On the other hand, ISS was defined as having a height with two standard deviations below the mean height for age and gender, as well as these patients had a growth hormone concentration of more than 10 ng/ml at one occasion. The two group subjects received treatment with RGHT for one year. Patients with chromosomal abnormalities, organic causes of short stature, and patients with familial short stature were excluded from the study.

The following body parameters were taken at the first visit before the start of the treatment: chronological age, bone age, height, weight, body mass index (BMI), insulin-like growth factor (IGF-1), GH test, and growth velocity (GV).

The mean and standard deviation represented the continuous data, while percentage and frequency were used to represent the qualitative variables. Moreover, the chi-square test and t-test indicated the normally distributed data. Categorical data were indicated as numbers. A p-value of less than 0.05 was considered statistically significant. Statistical package for the social sciences (SPSS; IBM Corp., Armonk, NY) was used for data analysis.

## Results

Table 1 compares the two groups after growth hormone treatment.

**Table 1:** Comparison between GHD patients and ISS patients after GH treatment Values are presented as mean. GHD: growth hormone deficiency, ISS: idiopathic short stature, BMI: body mass index, IGF-1: insulin-like growth factor-1, BA: bone age

	ISS n=19	GHD n=36
At First Visit		
Age	10.91±2.749	10.7±2.38
Gender	Male 9, Female 10	Male 20, Female 16
Height (cm)	125.51±10.94	125.26±12.27
Weight (kg)	25.48±7.41	27.34±4.44
BMI (kg/m^2^)	16.2±2.43	17.7±3.56
GH test (ng/ml)	14.24±4.69	4.66±2.62
IGF-1 (ng/ml)	145.57±108.97	141.1±127.03
BA (yr)	8.92±3.64	6.89±2.86
After 1-Year of Treatment		
Height (cm)	134.04±10.90	134.231±12.88
Weight (kg)	29.5139	33.2892
GH dose (mg)	1.1±0.46	1.21±0.49
IGF-1 (ng/ml)	268.56	308.24

The total number of subjects was 55. Out of the 55 patients, 29 (52.7%) were males while 26 (47.3%) were females. The total number of patients with GHD was 36 (male 20, female 16). The total number of patients with ISS was 19 (male 9, female 10). The mean age for the total number of patients that participated in the study was 10.77±2.48 (GHD 10.7±2.38, ISS 10.91±2.74). Although the mean age values for both groups were different from one another; the t-test showed no significant difference. The mean initial height for the total number of subjects was 125.35±11.72 cm (GHD 125.26±12.27 cm and ISS 125.51±10.94 cm). The mean initial body weight measured in kilogram (kg) was 27.34±4.44 for patients with GHD and 25.48±7.41 for patients with ISS. The mean BMI for patients with GHD was 17.7±3.56 kg/m^2^, and 16.2±2.43 kg/m^2^ for patients with ISS. The results for the mean growth hormone concentration after the growth hormone-stimulation test were much lower in patients with GHD than in ISS. The values for the mean growth hormone concentration were 4.66±2.62 ng/ml and 14.24±4.69 ng/ml for patients with GHD and ISS, respectively. The mean initial IGF-1 concentration was 141.1±127.03 ng/ml for patients with GHD and 145.57±108.97 ng/ml for patients with ISS. The mean initial bone age for patients with GHD was lower than that of ISS (6.89±2.86 yrs. vs. 8.92±3.64 yrs.). After one year of treatment with RHGT, the height, weight, and BMI were monitored. The average daily dose of GH was 1.21±0.49 mg and 1.1±0.46 mg for patients with GHD and ISS, respectively. Both groups were using the medication six days a week for one year.

**Figure 1:**
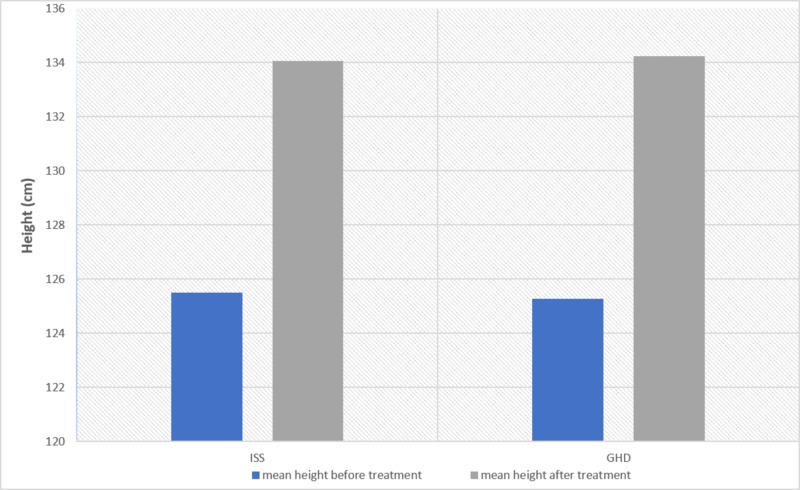
Mean height gain after one year of treatment for patients with GHD and ISS GHD: growth hormone deficiency, ISS: idiopathic short stature

 The mean height of both groups showed a significant increase after the treatment. GHD patients achieved a mean height of 134.231±12.88 as compared to their mean height before the treatment (134.231±12.88, 125.26±12.27, P=0.000). ISS patients achieved a mean height of 134.04±10.90 cm as compared to the mean height before the treatment (134.04±10.90, 125.51±10.94, P=0.000). However, when comparing the height gain after the treatment between the two groups, the values of the mean height gain showed no significant difference (134.231±12.88, 134.04±10.90 cm, P= 0.437) (Figure 1). The weight and BMI values were statistically insignificant.

## Discussion

Growth hormone treatment was initially provided to patients from animal sources. However, the development of some life-threatening side effects encouraged scientists to seek an alternative, safer method of treatment. In 1985, the first recombinant human growth hormone (rhGH) was approved by the Food and Drug Administration (FDA). This revolutionary change in the availability and safety of the drug has led to the extension in its use. Through the past 40 years, rhGH has been used in a variety of medical conditions, including Turner syndrome, chronic renal insufficiency, short stature, intra-uterine growth retardation, and growth hormone deficiency. Treatment with growth hormone has been strongly established in multiple studies to effectively increase the growth velocity and achieve the normal final adult height in children with short stature [1-2]. In addition, the term short stature is referred clinically to the diagnosis of height that is two standard deviations below the mean or average height for age and gender of the same population. Some causes of short stature have been linked to genetic factors while others could be due to an organic insult resulting in interference with any of the processes responsible for the growth and development of an individual. However, most cases of short stature are due to an unclear cause. Children presenting with short stature, which has not been linked to any apparent chromosomal abnormalities or genetic factors, are usually diagnosed as having either idiopathic short stature or constitutional growth delay. However, these two terms are often used interchangeably [3]. Growth hormone treatment was mostly used in the treatment of short stature due to GHD until 2003 where it was approved to be used as well in the treatment of ISS [4]. GHD is increasingly seen in the pediatric patient population these days. This can be attributable to the increased incidence of brain tumors and the utilization of different treatment modalities, including chemotherapy, radiation therapy, and surgery, which resulted in a potential expected side effect such as GHD [5-6].

Even though the use of recombinant growth hormone has been widely established in the treatment of GHD due to various causes, the indication for its use, and the effectiveness of the drug in patients with ISS remains controversial. However, the United States of America has approved its use in ISS due to the fact, these patients might suffer some psychosocial sequelae [7-8]. In addition, some studies have found that the use of rhGH in treating patients with ISS who are not suffering significant psychosocial burden was not associated with a better performance in outcomes in various life aspects [9]. Many studies have shown that the use of rhGH can improve height gain and growth velocity in patients with ISS treated for a short period of time. However, the use of this medication in ISS might result in a rapid increase in bone age and early closure of the epiphyseal plate, which could eventually affect these patients’ ability to attain expected adult height [9-13]. In contrast, many published research papers documented the efficacy of using rhGH in increasing the expected final adult height and growth velocity in patients with ISS [14-24]. In our study, we aimed mainly to compare the effect on height in patients with GHD and ISS after one year of treatment with rhGH. The mean height values of both groups were not significantly different at the time of diagnosis. Even though the mean height of both groups increased significantly compared to those prior to the treatment, the two values remained insignificant when compared with each other, which is consistent with most of the clinical trials. Despite that, patients with ISS showed a slightly higher change in mean height compared to those with GHD in the short-term treatment, which is also consistent with many clinical trials. The mean daily GH dose values of both groups were quite similar.so conclusion about whether the effects observed in these patients were dose-dependent could not be made. Side effects and adverse drug reactions were not reported during treatment except for two cases of mild gynecomastia, which could be attributable to physiological pubertal changes. However, some patients reported at least one attack of hypoglycemia usually in a period of eight to 12 hours following the injection. Fasting lipid profile and IGF-1 levels were closely monitored and kept within the normal range.

## Conclusions

 It appears from the study, the use of rhGH can improve growth velocity in ISS patients as effective as in GHD patients in a short-term period. From the fact that modern medicine emphasizes on the consideration of the biopsychosocial concept in managing diseases, the use of rhGH can possibly improve the psychosocial burden of the disease, especially in those with severe short stature. However, the cost-effectiveness of the treatment should not only focus on the biological effects of these patients but also the psychosocial outcomes of the treatment. In addition, the use of rhGH as a daily subcutaneous injection remains an issue and could affect compliance and adherence to the treatment. Some of the limitations in the study were the inability to find a sufficient number of patients who continued the treatment for a longer period of time, the inability to include a larger number of patients from the retrospective study due to missing data, and failure to make conclusions about some parameters because of the missing data. Larger sample size and a longer period of treatment are needed to accurately make conclusions about the use of rhGH in patients with GHD and ISS.
